# Eight years later: outcomes of CBT-treated versus untreated anxious children

**DOI:** 10.1002/brb3.274

**Published:** 2014-08-19

**Authors:** Gili W Adler Nevo, David Avery, Lisa Fiksenbaum, Alex Kiss, Sandra Mendlowitz, Suneeta Monga, Katharina Manassis

**Affiliations:** 1 University of TorontoToronto, ON, Canada; 2 Department of Psychiatry, Sunnybrook Health Sciences CentreToronto, ON, Canada; 3 Department of Psychiatry, The Hospital for Sick ChildrenToronto, ON, Canada; 4 York UniversityToronto, ON, Canada

**Keywords:** Adolescents, anxiety, children, cognitive–behavioral therapy, long-term follow-up, youth

## Abstract

**Background:**

Anxiety disorders are the most common psychiatric disorders of childhood, generate significant distress, are considered precursors to diverse psychiatric disorders, and lead to poor social and employment outcomes in adulthood. Although childhood anxiety has a significant impact on a child's developmental trajectory, only a handful of studies examined the long-term impact of treatment and none included a control group. The aim of this study was to conduct a long-term follow-up (LTFU) of anxious children who were treated with Cognitive–Behavioral Therapy (CBT) compared to a matched group of children who were not.

**Methods:**

Subjects comprised 120 children: a treatment group which included the first 60 consecutive consenting children who were diagnosed with an anxiety disorder and treated with CBT between the years 1997 and 2003 and a control group, 60 matched children who were assessed but not treated with CBT. An “ex-post-facto” design was used to compare the two groups.

**Results:**

Children showed lower rates of anxiety diagnosis (about 50% for both groups) and significantly improved functioning at LTFU (time effect *P* < 0.0001; no group difference). Anxiety levels were significantly lower in the nontreatment group at LTFU as compared to initial assessment (*P* = 0.02), but not in the treatment group, and a significant between-group difference was found (*P* = 0.01) according to child. An inverse relationship was found between self-efficacy/self-esteem and anxiety outcome ([*P* = 0.0008] and [*P* = 0.04], respectively).

**Conclusions:**

This study supports the assumption that childhood anxiety disorders may improve without treatment and highlights self-efficacy/self-esteem as potential factors in recovery.

## Introduction

Anxiety disorders are the most common psychiatric disorders of childhood, occurring in 5–20% of children (Bernstein et al. 1996; Esbjorn et al. 2010). They generate significant distress in the individual and an economic burden to society (Greenberg et al. 1999). Anxiety in children affects several domains critical to development, such as academic and interpersonal functioning (Pine 1997) which, in turn, lead to poor social and employment outcomes in adulthood (Duchesne et al. 2008; Woodward and Fergusson 2001; Kessler et al. 1997, 1995). Moreover, anxiety disorders are considered precursors to diverse psychiatric disorders including depression, bipolar disorder, psychosis, and drug abuse and dependence (Kendall and Kessler 2002; Last et al. 1997; Beesdo et al. 2010).

Cognitive–Behavioral Therapy (CBT) is considered a first-line, evidence-based treatment for childhood anxiety disorders (Silverman et al. 2008; In-Albon and Schneider 2007). One might expect a treatment that facilitates overcoming anxiety and addresses self-derogating cognitive attributions—CBT—would help the child alleviate immediate distress as well as influence her/his developmental trajectory. Although intuitively the short- and long-term benefits of CBT are clear, the soundness of evidence remains somewhat controversial. While some believe that evidence for short-term efficacy is strong and consistent (Silverman et al. 2008; In-Albon and Schneider 2007), others believe it is lacking with remission rates standing only at about 50% (James et al. 2005) and lack of evidence for the benefit of CBT over other therapeutic modalities (James et al. 2005). Although LTFU's are difficult to conduct (Toner and Stueve 1994), they are becoming more prevalent in recent years (Nevo and Manassis 2009; Beidel et al. 2006; Benjamin et al. 2013; Cobham et al. 2010; Ginsburg et al. 2014; McHugh O'Leary EM 2014; Saavedra et al. 2010; Watanabe et al. 2010). Findings from these studies indicate that initial treatment response is largely maintained over time. The largest study to date (Ginsburg et al. 2014) found that almost half of the sample (46.5%) were in remission at LTFU but outcome was unrelated to treatment arm (CBT, medication or CBT medication combination). The most problematic limitation of these studies is the inability to randomize subjects to a long-term control condition due to ethical considerations. The inability to include a control condition prohibits causal associations between treatment and outcome; unmeasured confounding variables (e.g., effects of maturation) could provide alternative explanations.

In this study, we tried to fill this gap addressing, in particular, the inclusion of a control group over a long period of time by utilizing available data from a long-standing, specialized anxiety disorders clinic for children. To this end, we used an “ex post facto” design to compare a group of anxiety-disordered children who were treated with CBT with a group of similar children who did not receive CBT approximately 8 years after they were assessed for treatment. We hypothesized that those who had received CBT as children would show fewer anxiety symptoms and anxiety diagnoses than those who had not (primary outcomes) and also show lower levels of depressive symptoms, fewer emerging disorders (such as affective disorders and substance abuse), and higher global functioning, self-efficacy, well-being, and self-esteem (secondary outcomes). We also explored potential predictors of long-term outcome, but with no a priori hypotheses.

## Materials and Methods

### Participants

Participants (*N* = 120) were 8–12 years old in 1997–2003, at the time of initial assessment at an academic facility specializing in pediatric anxiety. All subjects were diagnosed with a DSM primary anxiety disorder as ascertained by semistructured interview. Interviews were conducted by two child psychiatrists and one child psychologist, all of whom had extensive training and experience with the interviews. All subjects completed self-report anxiety, depression, and demographic questionnaires at the time of initial assessment.

Subjects comprised 120 children: a treatment group which included the first 60 consecutive consenting children who were treated with Cognitive–Behavioral Therapy (CBT) following initial assessment and a control group, 60 matched children (in age, gender, diagnosis and clinical severity at the time of assessment) who were assessed but not treated with CBT. All patients seen at our pediatric anxiety disorders clinic during the duration of the study were included and so the sample was representative of our clinic's population. A random draw was used in the event of more than one exact match. Controls were not treated with CBT at our clinic for a variety of reasons (e.g., geographic distance, scheduling difficulties, family not interested, etc.). Participants who received some form of CBT in the community at any stage of LTFU were excluded (i.e., participants received either no intervention or “treatment as usual” in the community. Treatment as usual comprised supportive therapy, religious or spiritual guidance and/or medication). Comorbid disorders were permitted. The only exclusion criterion was a severe physical or mental disability, which would render the participant unable to complete measures.

All study procedures were approved by the hospital IRB. No adverse effects were documented.

### Intervention

Treated children received CBT consisting of 12 sessions of group or individual therapy with 12 concurrent parent sessions, using the “Coping Bear” manual (group and Individual therapy were found to be equally efficacious (Silverman et al. 2008)). This manual is a previously evaluated local adaptation of Kendall's “Coping Cat” protocol for childhood anxiety disorders (Manassis et al. 2002). All therapists were extensively trained in CBT.

### Follow-up procedure

**Figure 1:**
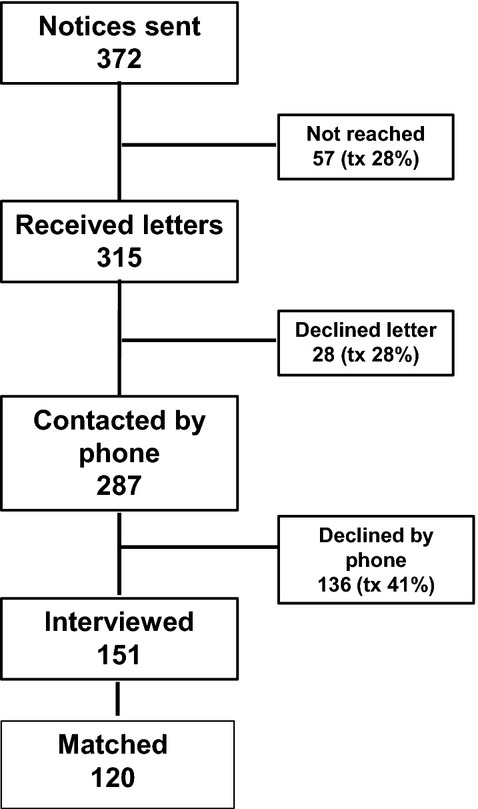
Points of attrition.

A letter was sent to all families who were assessed at the anxiety disorders program in the years 1997–2003 informing them of the study and asking them to contact the clinic if they did not wish to be approached. Families who agreed were contacted by telephone, detailed explanations regarding the study were given, and patients and their parents were asked for verbal consent. Consenting families were asked five preliminary questions (including utilization of any type of treatment up until the time of the interview) at the time of initial telephone conversation (script available upon request) and scheduled for a 2- to 3-h interview with the patient and 20- to 30-min interview with their parent (most often the mother). Written consents were acquired at this stage. All interviews were conducted at the participants' convenience, mostly face-to-face either in their home or at the clinic's offices, during evenings or weekends if necessary. On seven occasions, due to the patient's residence at University or move abroad, the interview was conducted over the telephone. Four psychology trainees (one BA, one MA, and two PhD candidates), trained in administering the ADIS semistructured interview and all measures used in the study, conducted the interviews. Interviewers were blind to group and distributed evenly between the treatment and control groups. See Fig. 1 for process flow.

### Outcome assessment

Information was obtained from subjects and their parents. Our general guidelines were to obtain information from subjects regarding current symptoms and from parents when knowledge about the family or long-term issues were needed, and also to corroborate primary outcomes (Mokros et al. 1987; Sawyer et al. 1992). See Table 1 for measure distribution.

**Table 1:** Measures and their administration

Domain		Initial assessment		LTFU	
			
Informant	Measure	Parent	Child	Parent	Child
Diagnostic information	ADIS	Clinician administered	Clinician administered	Clinician administered	Clinician administered
	DICA	Clinician administered			
Demographic information	Family and Household Survey Form: Ontario Child Health Study	Self-report		Self-report	
Anxiety	MASC (primary outcome measure)	Self-report about child	Self-report	Self-report about child	Self-report
	RCMAS	Self-report about child	Self-report		
	SCARED	Self-report about child	Self-report		
	SASC-R	Self-report about child	Self-report		
Depression	CDI		Self-report		Self-report
	BDI				Self-report
Functioning and Related measures	CGAS	Clinician rated		Clinician rated	
	GAF			Clinician rated	
	Personal Well-being Index				Self-report
	General Self-Efficacy scale				Self-report
	Rosenberg Self-Esteem Scale				Self-report
	Brief Family assessment measure	Self-report about family	Self-report about family	Self-report	Self-report
Parent symptoms	BSI			Self-report	
Improvement	CGI			Clinician rated	

Demographic information was obtained through the Ontario Child Health Study Family and Household Survey Form (Boyle et al. 1987).

#### Primary outcomes

We used the *Anxiety Disorders Interview Schedule (ADIS) for DSM-IV-TR* child version (Albano and Silverman 1996) to establish diagnosis, categorical primary outcome, comorbidity, and substance use. *The Diagnostic Interview for Children and Adolescents (DICA)* (Herjanic and Reich 1997) was used to establish diagnosis in 36 children at initial assessment. The use of more than one semistructured interview is due to the study's design which utilizes existing data. The dimensional primary outcome was anxiety severity as measured by *The Multidimensional Anxiety Scale for Children (MASC)* (Baldwin and Dadds 2007*;* March et al. 1997) at LTFU and for most initial assessments. Forty-two children were assessed for anxiety level at initial assessment using the *Revised Children's Manifest Anxiety Scale (RCMAS)* (Reynolds and Richmond 1985), *Screen for Child Anxiety Related Emotional Disorders (SCARED)* (Birmaher et al. 1997), and *The Social Anxiety Scale for Children—Revised (SASC-R)* (La Greca and Stone 1993).

#### Secondary outcomes

The *Children's Depression Inventory (CDI)* (Kovacs 1981*;* Smucker et al. 1986) was used to assess depression in youth younger than 17 and the *Beck Depression Inventory (BDI)* (Beck et al. 1988) for youth older than 17. The BDI has been normed for use with adolescents. Functioning was assessed by the interviewers using information from the ADIS. Depending on child age, either the *Global assessment of Functioning (GAF) Scale* (Endicott et al. 1976) or the *Children's Global Assessment Scale (CGAS)* (Shaffer et al. 1983) was used. The GAF scale, based on a continuum of mental health and mental illness, is a 100-point scale. Functioning is measured in three major areas: social functioning, occupational functioning, and psychological functioning. The CGAS is an adaptation of the GAF for children aged 4–16 years. Other measures related to functioning were *The Personal Well-being Index (Adult and School Children) (PWI-A and PWI-SC)* (Cummins et al. 2003) which contains seven items of satisfaction, each one corresponding to a quality of life domain (e.g., achievement, relationships). The school children and adult version were used, according to the participants' age; *The General Self-Efficacy Scale (GSE)* (Schwarzer and Jerusalem 1995). Perceived self-efficacy is the belief that one can perform a novel or difficult task, or cope with adversity in various domains of functioning. Negative coefficients were found with depression, anxiety, stress, burnout, and health complaints; *The Rosenberg Self-Esteem Scale (SES)* (Crandal 1973*;* Rosenberg 1965). The Rosenberg Self-Esteem Scale has demonstrated good reliability and validity across a large number of different sample groups, including adolescents and adults.

#### Potential outcome predictors

The *Brief Symptom Inventory-53* (Derogatis 1975) was used to assess psychological symptoms (e.g., depression, anxiety, and somatization) in parents. The measure correlates highly with clinical ratings and also differentiates between patient and nonpatient groups. The *Brief Family Assessment Measure (FAM)* (Skinner et al. 1995) was used to assess family functioning. Adverse life events were measured using the Coddington Life Events Scale for Adolescents (CLES-A, Coddington 1999). Both desirable and undesirable life events are incorporated in this scale and norms are provided according to age group. The 75th percentile is used as a cutoff point for identifying at risk individuals.

### Data analysis

Descriptive statistics were calculated for all variables of interest. Continuous measures such as age were summarized using means and standard deviations, whereas categorical measures were summarized using counts and percentages.

Given that four different measures were used for anxiety on initial assessment (RCMAS, SCARED, MASC, and SASC-R), each measure was standardized to a distribution with mean 0 and standard deviation of 1. A mean score was then calculated for each subject for whichever measures that subject completed and this represented their ‘pre’ anxiety score. Within-group comparisons of pre to post anxiety were carried out using paired t-tests, whereas between-group comparisons at the different time points were carried out using two sample t-tests. Linear regression models were run to assess changes between groups at LTFU controlling for baseline scores Categorical outcomes were compared between groups using chi-square analyses. All analyses were carried out using SAS Version 9.2 (SAS Institute, Cary, NC).

## Results

### Group differences and attrition

Thirty-two percent of patients who were initially assessed and diagnosed at our clinic with a DSM anxiety disorder participated in Long-Term Follow-Up (LTFU) (see Fig. 1 for details). Although a higher proportion of nontreatment than treatment subjects declined participation in the study, participants and nonparticipants did not differ significantly in pretreatment socio-demographic characteristics (i.e., age, gender, ethnicity, household income, parent level of education), and pretreatment clinical variables (diagnoses, degree of clinical severity). The majority of participants in both groups (91.6% in the treatment group and 95% in the nontreatment group) were diagnosed with Separation, Generalized, or Social Anxiety disorders. These diagnoses are often grouped together in clinical studies due to a high degree of overlap in symptoms and their distinction from other anxiety disorders. Remaining participants were diagnosed with Panic Disorder, simple phobia, OCD, and Anxiety NOS. No significant differences in socio-demographic characteristics or clinical variables were found between treatment and nontreatment participants. In particular, anxiety symptom levels (primary outcome) did not differ significantly between groups. See Table 2 for details.

**Table 2:** Demographic characteristics and pretreatment measures

	Tx Mean ± SD	Non-Tx Mean ± SD	*P*
Age	9.6 ± 1.2	9.6 ± 1.3	0.91
Time Elapsed since Initial Evaluation	8.0 ± 1.7	8.4 ± 1.5	0.14
Gender (Female) %	53.5	41.4	0.19
SES	53 ± 15.4	48.1 ± 10.9	0.09
Comorbid disorders %	46	48	0.77
Comorbid externalizing %	13	10	0.51
Anxiety level (child standardized)	−0.04	0.22	0.13
Anxiety level (parent standardized)	0.03	0.01	0.93
Depression (CDI)	47.2 ± 8.5	49.2 ± 12.6	0.36
Functioning (CGAS)	55.8 ± 7	56 ± 7.3	0.82
Family functioning (Brief FAM)	65.8 ± 10	68.6 ± 5.5	0.08

### Diagnostic status

Diagnostic status was assessed through a semistructured interview conducted by a trained interviewer at all time points. All participants in the study met criteria for at least one DSM anxiety diagnosis on initial assessment. Approximately half of participants (50% in the treatment group and 48.1% in the nontreatment group) did not meet criteria for an anxiety diagnosis at LTFU. No significant between-group difference was found (chi sq=0.0418; *P* = 0.84). Remaining participants were diagnosed with the same diagnosis (22.6% in the treatment group vs. 26.9% in the nontreatment group), different anxiety diagnosis (38.7% in the treatment group vs. 46.2% in the nontreatment group), or a nonanxiety DSM diagnosis (22.6% in the treatment group vs. 26.9% in the nontreatment group). Some had multiple diagnoses. No significant between-group difference was found for any of these categories (chi sq=1.57, *P* = 0.21; χ^2^ = 0.64, *P* = 0.42; and χ^2^ = 0.29, *P* = 0.59, respectively). Interestingly, very few participants were diagnosed with affective disorders or substance abuse/dependence, no significant between-group difference was found (*P* = 0.54 and 0.64, respectively). See Table 3 for details.

**Table 3:** Diagnoses at Initial Interview and at LTFU

	Participants, *N* (% of group)							
	
	Initial interview				LTFU			
		
	CBT		Non-CBT		CBT		Non-CBT	
Diagnosis	Primary	Secondary	Primary	Secondary	Primary	Secondary	Primary	Secondary
separation anxiety	8 (13)	4 (7)	16 (27)	4 (7)	1 (2)	0	0	0
Generalized anxiety disorder	38 (63)	6 (10)	35 (58)	7 (12)	8 (13)	2 (3)	11 (18)	5 (8)
Social anxiety disorder	9 (15)	4 (7)	6 (10)	4 (7)	6 (10)	0	11 (18)	5 (8)
Simple phobia	3 (5)	4 (7)	0	9 (15)	0	0	0	0
Obsessive–compulsive disorder	1 (2)	1 (2)	1 (2)	2 (3)	1 (2)	1 (2)	1 (2)	2 (3)
Panic disorder	0	1 (2)	1 (2)	2 (3)	4 (7)	0	2 (3)	2 (3)
Depression	0	0	0	1 (2)	0	0	0	0
Attention-deficit/hyperactivity disorder	0	2 (3)	0	3 (5)	3 (5)	0	3 (5)	4 (7)
Oppositional defiant disorder	0	2 (3)	0	3 (5)	0	0	0	0
Anxiety NOS	1 (2)	0	1 (2)	0	0	0	0	0
Agoraphobia	0	0	0	0	3 (5)	0	1 (2)	0
Posttraumatic stress disorder	0	0	0	0	0	0	1 (2)	1 (2)
Other	0	0	0	0	1 (2)	0	0	2 (3)
Marijuana infrequent/alcohol misuse						7 (12)		5 (8)
Alcohol or drug abuse or dependence					3 (5)		2 (3)	

### Symptom severity

#### Anxiety

**Figure 2:**
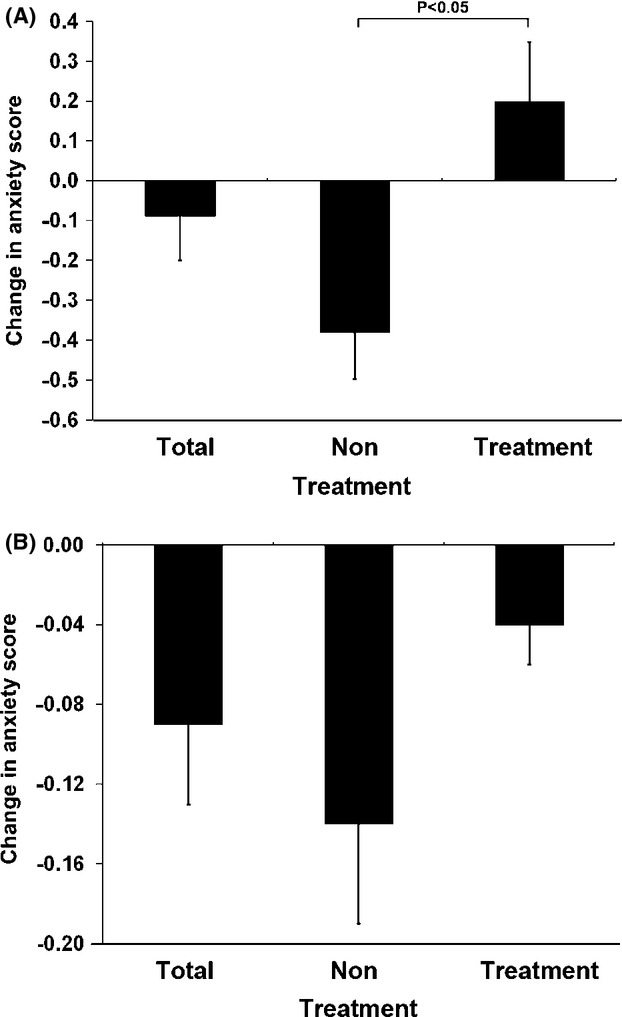
Pre-post anxiety levels. Change in standardized anxiety levels from baseline according to child (A) and parent (B) measures.

A statistically significant decrease in total anxiety was found in the nontreatment group at LTFU according to child measures (0.38 SD decrease in anxiety; *P* = 0.02), while an insignificant increase in anxiety was found in the treatment group (0.2 SD increase in anxiety; *P* = 0.06). The difference between treatment and nontreatment groups at LTFU, according to child measures, was significant (*P* = 0.01). Anxiety levels for the group, taken as a whole, were lower at LTFU by 0.088 SD, but the difference was not significant (*P* = 0.44). The subcategories of harm avoidance and social anxiety showed a significant between-group difference with the nontreatment group having a lower mean than the treatment group (*P* = 0.041 and *P* = 0.04, respectively). No significant difference was found on other MASC subcategories, namely, physical symptoms and separation/panic. Parents' perception of their child's anxiety presented no significant differences in treatment, nontreatment, and the group taken as a whole at LTFU as compared to initial assessment (*P* = 0.84, 0.55 and 0.55, respectively). No difference between groups was found at LTFU according to parents (*P* = 0.41) (Fig. 2).

#### Depression

Initial depression level as measured by child CDI on assessment was within normal range (8.82 on average) without significant difference between groups (*F*(1,107)=0.02, *P* = 0.88). No significant difference between groups (as measured by the BDI) controlling for baseline depression was found at follow-up as well (F (1,97)=0.06, *P* = 0.81).

#### Involvement in life

##### Functioning

The mean level of functioning on initial assessment as determined by the CGAS measure was 55.92 (“Variable functioning with sporadic difficulties or symptoms in several but not all social areas; disturbance would be apparent to those who encounter the child in a dysfunctional setting or time but not to those who see the child in other settings.”). No significant difference between groups was found (*F*(1,117)=0.89, *P* = 0.35). The mean level of functioning at LTFU as determined by a composite CGAS/GAF score was 72.75 (“No more than slight impairments in functioning at home, at school, or with peers; some disturbance of behavior or emotional distress may be present in response to life stresses.”). Again, no significant difference between groups was found (*F*(1,117)=2.93, *P* = 0.09). The improvement in functioning at LTFU as compared to initial assessment was found to be significant (*P* < 0.0001).

##### Quality of life, self-esteem and self-efficacy

The personal well-being index was used to measure quality of life, the general self-efficacy scale was used to measure self-efficacy, and the Rosenberg self-esteem scale was used to measure self-esteem. These measures were not given to patients at initial assessment hence pre-post analysis was not possible. All three variables (quality of life, self-esteem, and self-efficacy) were within normal range at follow-up (mean = 7.12 ± 1.8; 2.26 ± 0.66 and 3.08 ± 0.52, respectively). No significant between-group difference was found (*F*(1,114) = 0.6, *P* = 0.44; *F*(1,114)=1.07, *P* = 0.3 and *F*(1,114) = 0.22, *P* = 0.64, respectively).

### Potential outcome predictors

The dependant variable studied was anxiety level as measured by the MASC at LTFU. Our sample size allowed our linear regression model to fit a maximum of 12 predictors. The following independent variables were included in the analysis: group (tx/non-tx), SES status, age at initial assessment, level of functioning at initial assessment, quality of life, self-esteem, self-efficacy, family functioning, maternal anxiety, depression at initial assessment, and number of adverse life events. A significant inverse relationship was found between self-esteem (*B* = −4.08, *P* = 0.04) and self-efficacy (*B* = −10.2, *P* = 0.0008) as compared to anxiety outcome. Anxiety decreased as self-esteem and efficacy increased. No other independent variables showed a significant relationship with the dependant variable.

## Discussion

Long-Term Follow-Up (LTFU) studies are difficult to conduct and generally rare, but the interest in the LTFU of treated anxious children is just starting to emerge. This is the first long-term study (i.e., follow-up >2 years) to compare youth treated for their anxiety with CBT during childhood versus those who were not treated (thus controlling for confounders). Our results are surprising. Anxiety levels (according to child) were found to be significantly lower in the nontreatment group (a group of children who were not treated with CBT) at LTFU as compared to initial assessment, but anxiety levels in the treatment group were found to be insignificantly higher. Incidence of anxiety diagnosis was significantly lower in both groups, dropping to 50% of patients. In addition, the MASC subcategories of harm avoidance and social anxiety for the nontreatment group were significantly lower at LTFU according to child measures but not parental measures. Both groups showed a significant improvement in functioning at LTFU. Another significant finding was an inverse relationship between self-efficacy and self-esteem as compared to anxiety outcome for the whole group. Anxiety decreased as self-efficacy and esteem increased.

Our study is consistent with previous studies in remission rates (Ginsburg et al. 2014; James et al. 2013) (i.e., freedom from any anxiety diagnosis) but not in symptomatic relief (i.e., decrease in anxiety symptoms on continuous measures). Previous studies showed a significant decrease in anxiety symptoms in children who were treated with CBT at LTFU (Nevo and Manassis 2009; Ginsburg et al. 2014). This inconsistency may be explained by our study's limitations: The study's design was not a randomized controlled trial (due to obvious ethical considerations) but rather an ex post facto design, that is, one that utilizes naturally occurring groups for a prospective examination. Our study had a high attrition rate compared to other LTFU studies, which may have resulted in selection bias. The unique structure of an academic clinic allowed us to recruit a sufficient number of patients who had been assessed with a semistructured interview and had preexisting measures. However, this benefit also had its disadvantages. Our questionnaire battery was not set up with a long-term study in mind and new measures were regularly introduced to the clinic, leaving us with the difficult task of comparing several preexisting anxiety measures to the measure we introduced at follow-up—the MASC.

Nevertheless, the results offer a speculative glimpse at alternative interpretations of existing LTFU's for this population; the working hypothesis within the field has been that children do not outgrow their anxiety, that the majority of anxiety diagnoses do not remit spontaneously over time (Keller et al. 1992; Kendall and Ollendick 2004; Pine et al. 1998) but, surprisingly, little is known regarding the natural course of anxiety disorders. Some evidence suggests that more than 50% of the prevalent anxiety disorders remit spontaneously over time (Chartier et al. 1998). Our study provides tentative support for these findings since remission was not linked to group.

Pre-post analyses showed significantly lower overall anxiety (our primary outcome measure), harm avoidance, and social anxiety in the nontreatment group. This is puzzling. A possible explanation may be subtle differences, undetectable in our pretreatment statistical analysis, between the groups. Perhaps patients and their families who were less avoidant and socially phobic (attributes which prevailed over time) felt more comfortable coping with anxiety on their own without the support of a professional, resulting in self-selection to the control condition.

A significant inverse relationship was found between anxiety and self-efficacy and self-esteem at follow-up, that is, higher self-esteem and self-efficacy seem to be protective against anxiety symptoms. The inverse relationship between anxiety and self-esteem/self-efficacy is consistent with previous studies that show, not somatic arousal per se, but the meaning attributed to it and the belief that one has the power to cope with it, to be the differentiating factor between clinical and nonclinical groups (Telch et al. 1985; Vermilyea et al. 1984). To the best of our knowledge, this is the first study to provide evidence for this correlation in the context of therapy.

Although our study did not show the results we expected, that is, children in the treatment group faring better than those in the control condition at long term, we view its findings as positive. The study highlights two critical issues in the treatment and study of anxiety treatment in children and adolescents: The first is the importance of addressing not only anxiety symptoms but also self-efficacy and self-esteem when treating anxiety-disordered children. The second is the importance of undertaking LTFU's with control conditions in order to accurately assess long-term outcome. LTFU's can inform us of a variety of longitudinal courses which are easily missed in cross-sectional designs. They can inform us of potential subtypes of anxiety disorders, which can manifest in different courses and outcomes, and may require different treatment strategies.

## Conclusion

Our study is unique because we were able to follow untreated anxious children as compared to treated children over an average of 8 years in a naturalistic fashion utilizing existing data and infrastructure. Our findings, that show approximately 50% of anxious children remit over time, are consistent with previous LTFU findings, which followed only treated children (Ginsburg et al. 2014; James et al. 2013). Contrary to previous belief, CBT did not appear to enhance remission rates relative to our non-CBT control condition. Although these findings should be taken with a grain of salt due to inherent methodological shortcomings, we are hopeful that they would propel researchers in the field to address challenges posed by LTFU's and replicate this study. Our findings show a correlation between self-efficacy/self-esteem and positive outcome, regardless of anxiety severity. Future treatments may benefit from including interventions focusing on self-efficacy as well as anxiety symptoms.
